# CpxAR of Actinobacillus pleuropneumoniae Contributes to Heat Stress Response by Repressing Expression of Type IV Pilus Gene *apfA*

**DOI:** 10.1128/spectrum.02523-22

**Published:** 2022-10-19

**Authors:** Feng Liu, Wei Peng, Kang Yan, Jing Huang, Fangyan Yuan, Qigai He, Huanchun Chen, Weicheng Bei

**Affiliations:** a State Key Laboratory of Agricultural Microbiology, College of Veterinary Medicine, Huazhong Agricultural University, Wuhan, Hubei, China; b College of Animal Sciences, Yangtze University, Jingzhou, Hubei, China; c Hubei Hongshan Laboratory, Wuhan, China; d Key Laboratory of Prevention and Control Agents for Animal Bacteriosis (Ministry of Agriculture), Institute of Animal Husbandry and Veterinary Sciences, Hubei Academy of Agricultural Sciences, Wuhan, China; e School of Foreign Languages, Zhejiang Gongshang University, Hangzhou, Zhejiang, China; Texas A&M University

**Keywords:** *Actinobacillus pleuropneumoniae*, heat stress, TCS, CpxAR, type IV pilus

## Abstract

Acute pleuropneumonia in swine, caused by Actinobacillus pleuropneumoniae, is characterized by a high and sustained fever. Fever creates an adverse environment for many bacteria, leading to reduced bacterial proliferation; however, most pathogenic bacteria can tolerate higher temperatures. CpxAR is a two-component regulation system, ubiquitous among Gram-negative bacteria, which senses and responds to envelope alterations that are mostly associated with protein misfolding in the periplasm. Our previous study showed that CpxAR is necessary for the optimal growth of Actinobacillus pleuropneumoniae under heat stress. Here, we showed that mutation of the type IV pilin gene *apfA* rescued the growth defect of the *cpxAR* deletion strain under heat stress. RNA sequencing (RNA-seq) analyses revealed that 265 genes were differentially expressed in the Δ*cpxAR* strains grown at 42°C, including genes involved in type IV pilus biosynthesis. We also demonstrated direct binding of the CpxR protein to the promoter of the *apf* operon by an electrophoretic mobility shift assay and identified the binding site by a DNase I footprinting assay. In conclusion, our results revealed the important role of CpxAR in A. pleuropneumoniae resistance to heat stress by directly suppressing the expression of ApfA.

**IMPORTANCE** Heat acts as a danger signal for pathogens, especially those infecting mammalian hosts in whom fever indicates infection. However, some bacteria have evolved exquisite mechanisms to survive under heat stress. Studying the mechanism of resistance to heat stress is crucial to understanding the pathogenesis of A. pleuropneumoniae during the acute stage of infection. Our study revealed that CpxAR plays an important role in A. pleuropneumoniae resistance to heat stress by directly suppressing expression of the type IV pilin protein ApfA.

## INTRODUCTION

Porcine contagious pleuropneumonia, a contagious respiratory disease caused by Actinobacillus pleuropneumoniae, leads to significant economic losses in the global swine industry. The peracute form is a febrile systemic disease characterized by a high mortality rate and high fever (42°C). The fever response is a protective mechanism against invading pathogens, which resolves many infections, thereby aiding in survival. However, most Gram-negative bacteria, such as A. pleuropneumoniae, have evolved exquisite mechanisms to adapt to fever-like temperature stress to ensure their survival. They accomplish this adaptation by relying on signal transduction systems that modulate specific gene products that help the organism tolerate diverse environments.

Gram-negative bacteria contain a soluble compartment, known as the periplasm, which is located between the cytoplasmic (or inner) and outer membranes (1). A large number of nascent proteins that emerge from ribosomes are translocated through the Sec secretion pathway into this compartment in an unfolded conformation to perform important functions, such as the assembly of pili and flagella (2). Owing to the porous character of the outer membrane, the periplasm is particularly exposed to unfavorable environments (3). Environmental stress, such as elevated temperature, can cause nascent proteins to misfold, accumulate, and aggregate. Aggregated proteins pose a severe threat to cells, as they can cause lethal damage.

Gram-negative bacteria exhibit multiple envelope stress responses to protein misfolding in the periplasm and effectively respond to protein-folding stress. Among these, the Cpx systems were proposed to sense the presence of damaged periplasmic proteins and suppress the toxicity of misfolded proteins (4). The Cpx pathway is a typical two-component system (TCS) comprising a membrane-bound sensor kinase, CpxA, and a cytoplasmic response regulator, CpxR, that integrates different signals (5). These signals include elevated temperatures and overproduced periplasmic proteins, which might lead to misfolding of pilus subunits and β-barrel outer membrane proteins (6). Misfolded proteins induce CpxA activation, ultimately causing upregulated expression of the proteases and chaperones (DegP, DsbA, PpiA, and Spy) or downregulated expression of the misfolded proteins (7).

Type IV pili are found in many Gram-negative bacteria and are essential to surface motility, virulence, DNA uptake, biofilm formation, and autoaggregation (8). Our previous study showed that type IV pili play an important role in host cell adherence and biofilm formation in A. pleuropneumoniae (9). Furthermore, we have reported that the *apfABCD* (*apf*) and *pilMNOPQ* (*pil*) gene clusters are operons, and each gene in the *pil* and *apf* operons is necessary for Tfp biogenesis in A. pleuropneumoniae (9). Previous immunology and homology analysis confirmed that ApfA is the major pilin subunit in A. pleuropneumoniae (10). In Escherichia coli, pilus subunits were prone to misfolding and aggregation in the periplasm, leading to reduced bacterial survival under heat shock conditions (11).

Previously, we found that CpxAR is necessary for the optimal growth of A. pleuropneumoniae at 42°C (12). However, the mechanism of action of CpxAR in regulating A. pleuropneumoniae resistance to heat stress has not been fully elucidated. In this study, we tested the hypothesis that one of the mechanisms of this Cpx-mediated heat resistance is the downregulation of easily misfolded proteins. We showed that the Cpx response directly regulates the transcription of the gene encoding ApfA, which is involved in the mechanism of A. pleuropneumoniae resistance to heat stress. Taken together, these data demonstrate that the Cpx system contributes to A. pleuropneumoniae resistance to heat stress by directly suppressing the expression of ApfA.

## RESULTS

### The CpxAR two-component system regulates the expression of multiple genes.

**FIG 1:**
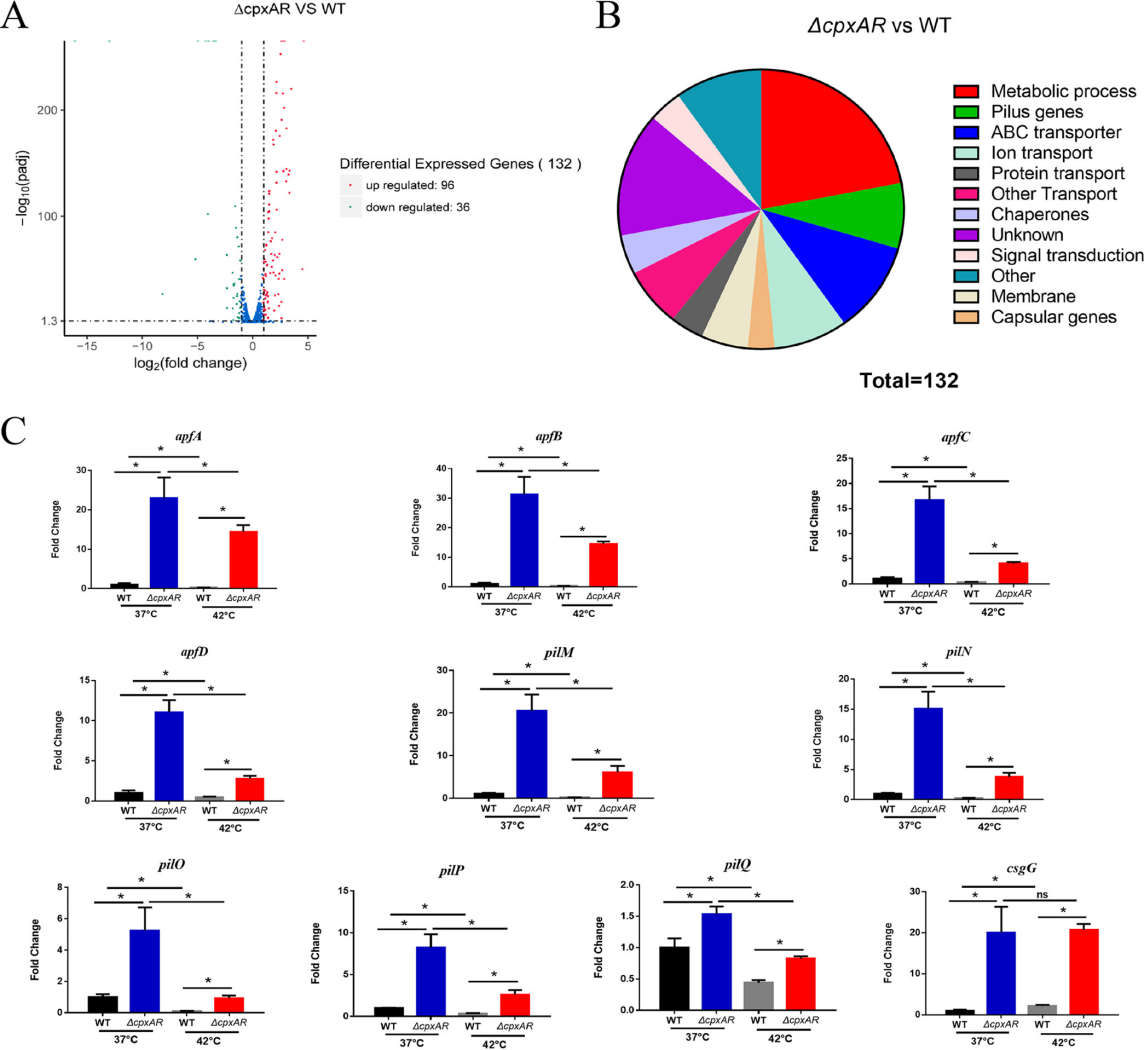
A. pleuropneumoniae CpxA/CpxR TCS represses pilus genes expression under heat stress. (A) Volcano plot showing the global changes in transcript levels of the Δ*cpxAR* mutant versus the WT strain grown at 42°C (log_2_ fold change, >1.0; log_2_ fold change, <–1.0; *P* < 0.05). (B) Gene ontology functional categories of the genes regulated by CpxA/CpxR. (C) The expression of pilus genes determined by RNA-seq was confirmed by RT-qPCR. The levels of gene expression were normalized to the housekeeping gene 16S rRNA. Means ± standard deviation (SD) from three independent experiments are shown. *, *P < *0.05.

To investigate the role of CpxAR in the fever-like temperature-resistance of A. pleuropneumoniae, we performed RNA-seq analysis to compare the transcriptome of the *cpxAR* mutant with that of the wild-type (WT) strain grown under heat stress (Gene Expression Omnibus [GEO] accession number GSE214078). Transcriptomic analysis identified 265 differentially expressed genes, 134 of which were upregulated in the Δ*cpxAR* mutant compared to the wild type at 42°C and of which 131 were downregulated (Fig. 1A).

To gain insight into the cellular pathways altered by loss of CpxAR, we classified differentially expressed genes into a variety of functional categories, such as metabolic process, pilus genes, ABC transporter, ion transport, capsular genes, membrane, protein transport, other transport, chaperones, signal transduction, and unknown function (Fig. 1B).

The pilus genes, including several type IV pilus-associated genes, such as *apfA*, *apfB*, *apfC*, *apfD*, *pilM*, *pilN*, *pilO*, *pilP*, and *pilQ*, were upregulated in the Δ*cpxAR* mutant compared with the WT strain at 42°C (Table 1), and this was confirmed by reverse transcription-quantitative PCR (qRT-PCR) (Fig. 1C). These genes were also upregulated at 37°C and 42°C except for the *pilQ* gene, as shown by qRT-PCR (Fig. 1C). In addition, these genes were downregulated at 42°C relative to 37°C in both WT and *cpxAR* mutant backgrounds (Fig. 1C). The data in Fig. 1C suggested that CpxAR was involved in repression of the pilus genes, independent of temperature. These observations indicated that there was a connection between A. pleuropneumoniae TCS CpxAR-mediated heat stress and the overexpression of pilus proteins.

**TABLE 1:** Pilus genes regulated by CpxA/CpxR

Gene no.	Gene name	Product	Fold change at 42°C
			(Δ*cpxAR* vs WT)
DRF63_RS04675	*apfA*	Type IV pilus protein	3.09
DRF63_RS04670	*apfB*	Type IV pilus protein	3.64
DRF63_RS04665	*apfC*	Type IV pilus protein	2.50
DRF63_RS04660	*apfD*	Type IV pilus protein	2.29
DRF63_RS01015	*pilM*	Type IV pilus protein	3.13
DRF63_RS01020	*pilN*	Type IV pilus protein	3.17
DRF63_RS01025	*pilO*	Type IV pilus protein	2.72
DRF63_RS01030	*pilP*	Type IV pilus protein	2.13
DRF63_RS01035	*pilQ*	Type IV pilus protein	1.33

### ApfA overexpression affects A. pleuropneumoniae resistance to heat stress.

**FIG 2:**
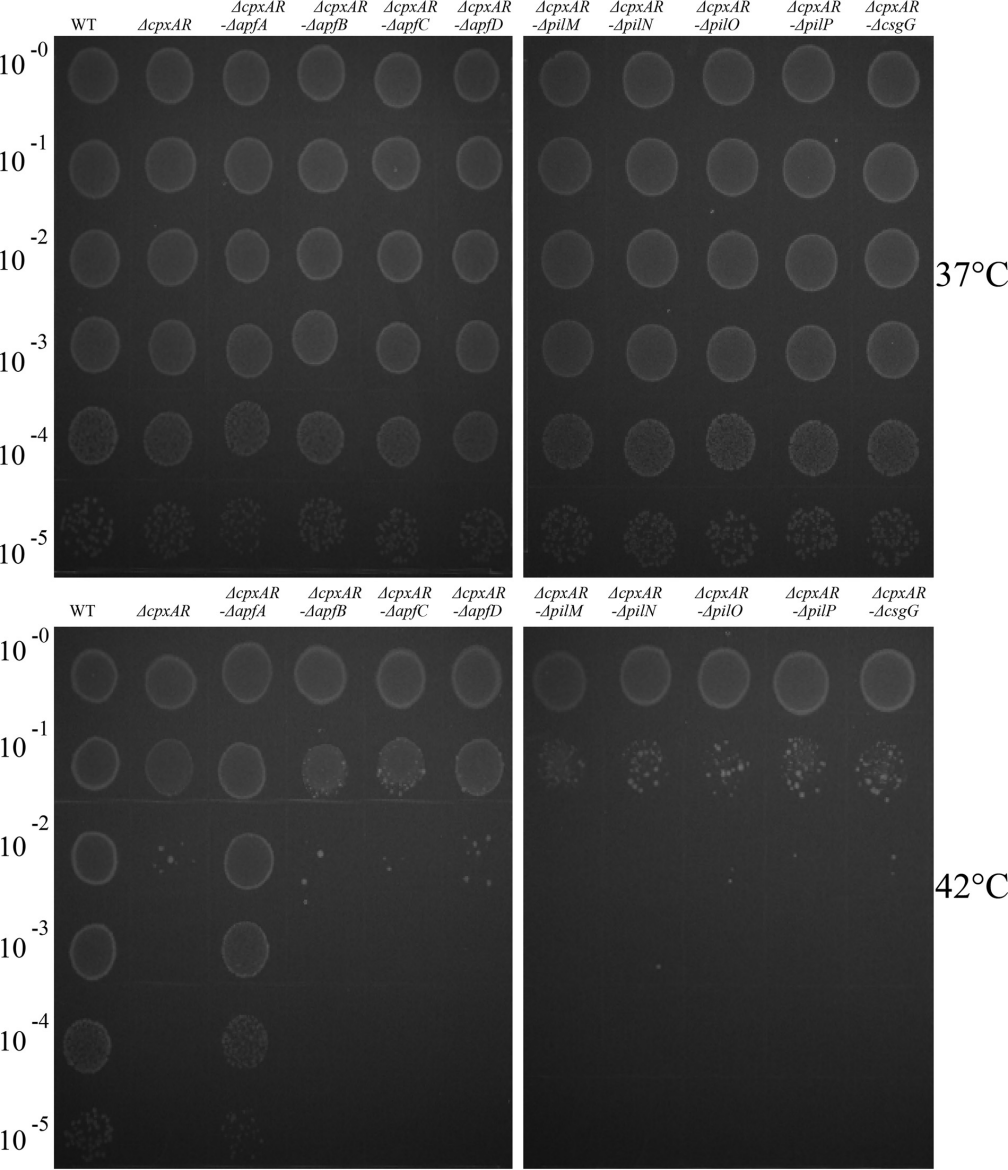
Role of the type IV pilus genes in bacterial growth under heat stress. The WT strain S4074 and its mutants were spotted on two TSA agar plates using 10-fold serial dilutions and incubated at 37°C and 42°C, respectively.

To examine whether the nine pilus genes (*apfA*, *apfB*, *apfC*, *apfD*, *pilM*, *pilN*, *pilO*, *pilP*, and *pilQ*) were crucial for A. pleuropneumoniae fever-like temperature-resistance, nine double mutant strains were constructed based on the Δ*cpxAR* mutant strain (see Fig. S1 in the supplemental material). The WT strain, the Δ*cpxAR* mutant, and the nine double mutants were grown at 37°C, and serial dilutions were spotted onto plates. All of the mutants displayed normal growth when incubated at 37°C (Fig. 2). However, when the plates were incubated at 42°C, only the Δ*cpxAR* Δ*apfA* mutant and the WT strain displayed normal growth, whereas the growth of other mutant strains was markedly reduced (Fig. 2).

**FIG 3:**
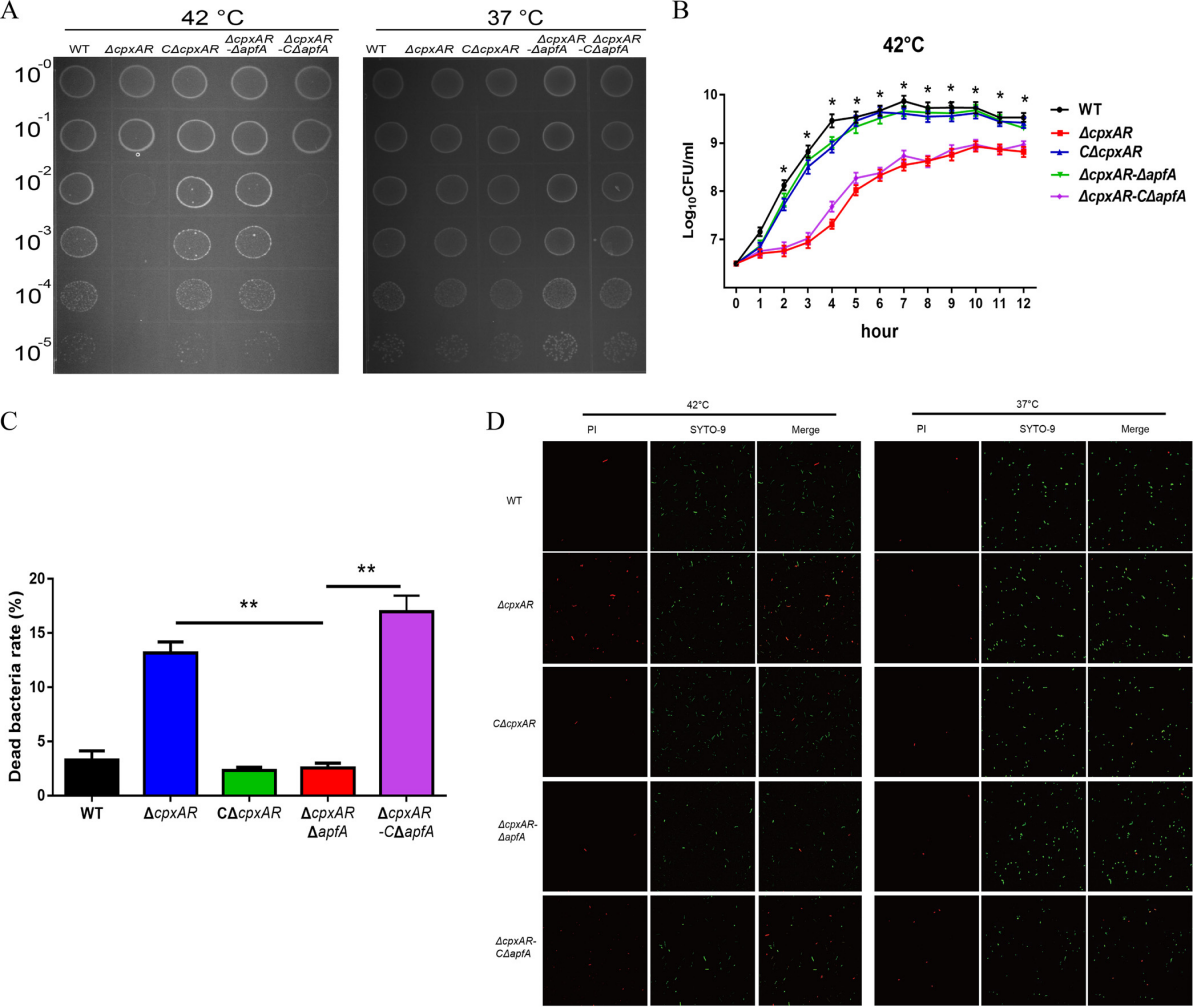
ApfA is involved in A. pleuropneumoniae responses to heat stress. (A and B) Bacterial growth under heat stress was monitored by spotting on TSB agar plates using 10-fold serial dilutions (A) and measurement of viable cell counts expressed as log CFU/mL (B). (C and D) Flow cytometric quantification (C) and fluorescence microscopy observation (D) of the viability of the bacterial population following treatment with heat stress. The live bacteria fluoresce green; dead bacteria are red. Results indicate the mean ± SD from three independent experiments. *, *P < *0.05; **, *P < *0.01.

To confirm the role of *apfA* in heat stress, we performed a complementation assay. Whether the cells were grown in solid or liquid medium, the growth of the complemented (C)Δ*cpxAR* strain was similar to that of the WT strain, and the growth defect in the Δ*cpxAR-*CΔ*apfA* strain was similar to that of the Δ*cpxAR* strain (Fig. 3A and B). The growth ability of these strains was confirmed by confocal microscopy and flow cytometry using the LIVE/DEAD BacLight kit and revealed that the number of propidium iodide-labeled bacteria was significantly decreased in the heat-stressed Δ*cpxAR* Δ*apfA* mutant, thus indicating lower death rates in this mutant compared with other strains under heat stress (Fig. 3C and D).

### CpxR represses ApfA by direct promoter binding.

**FIG 4:**
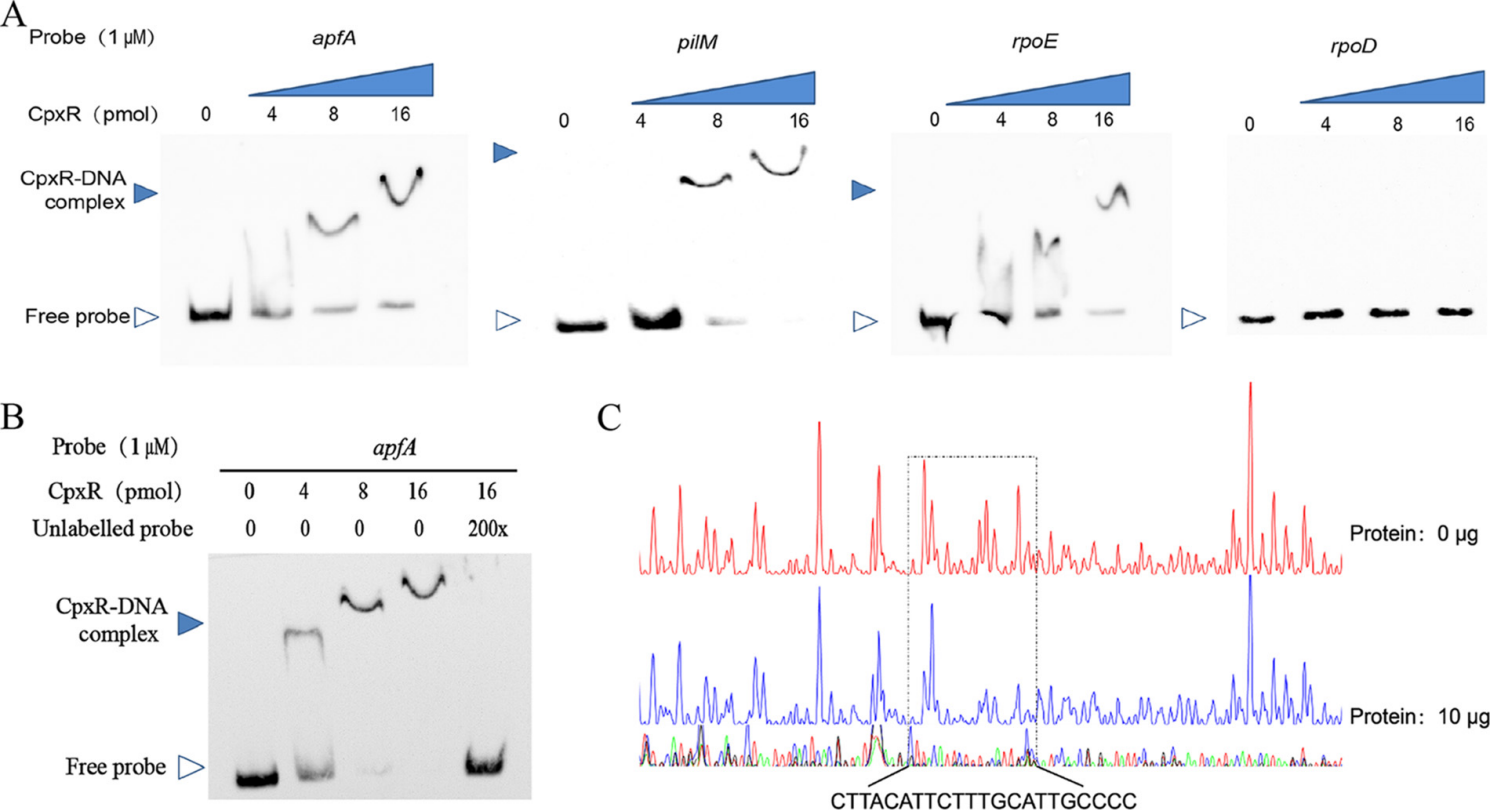
Identification of CpxR binding sites in the *apfA* promoter. (A) Electrophoretic mobility shift assays were used to analyze the direct binding of CpxR to the *apfA* and *pilM* promoter regions. *rpoE* and *rpoD* were, respectively, used as the positive and negative control. (B) Electrophoretic mobility shift assays were conducted to determine the impact of the unlabeled DNA probe on CpxR-*apfA* binding. (C) Footprinting analysis of CpxR binding to the *apfA* promoter. The nucleotide sequences protected by CpxR are shown below the electropherogram.

To test whether CpxR is able to bind to the promoter regions of *apfA* and *pilM*, electrophoretic mobility shift assays (EMSA) were performed. We observed concentration-dependent shifted bands for samples containing purified His-tagged CpxR and biotin-labeled *apfA*, *pilM*, and *rpoE* (positive control) promoter fragments (Fig. 4A). No shifted band was observed when the *rpoD* (negative control) promoter fragment was used, suggesting that the interaction of CpxR with the *apfA* and *pilM* promoter regions was specific. Competition by an unlabeled DNA probe attenuated the biotin signal of the CpxR-DNA complex, again supporting the view that the CpxR-*apfA* interaction was specific (Fig. 4B). In addition to EMSA, we performed a DNase I footprinting assay to map the precise binding sequence of CpxR to the FAM-labeled *apfA* promoter fragment. As shown in Fig. 4C, a 22-nucleotide (nt) region (3′-CTTACATTCTTTGCATTGCCCC-5′) from position −268 to −247 relative to the *apfA* translational start, was protected from DNase I digestion by CpxR binding.

## DISCUSSION

The two-component systems explored thus far control many biological processes in response to sudden changes in the environment, which is critical to successful infection (13). The CpxA/CpxR system is a pleiotropic TCS of E. coli required for envelope maintenance, biofilm formation, and pathogenesis (14). Our previous studies revealed that CpxA/CpxR is essential for A. pleuropneumoniae to grow in TSB broth at 42°C (15), suggesting that CpxA/CpxR is important in heat resistance for A. pleuropneumoniae. However, the underlying mechanism remained to be elucidated. In the present study, we identified a new CpxA/CpxR-regulated gene in A. pleuropneumoniae, namely, *apfA*. Furthermore, we showed that the CpxA/CpxR-ApfA pathway plays a crucial role in the heat stress response by A. pleuropneumoniae.

Under heat stress, proteins are prone to denature and misfold in the bacterial periplasm. The accumulation of damaged (misfolded) proteins, which are toxic, can lead to bacterial death. Bacteria have evolved elaborate quality control strategies to maintain proper cellular protein homeostasis. A series of studies revealed that the CpxA/CpxR system detects and responds to envelope stress by monitoring alterations in the envelope (16). The main outputs generated by the activation of Cpx are increased production of envelope chaperones and proteases, such as DegP, DsbA, and PpiA, to alleviate envelope stress by refolding and degrading misfolded proteins (11). Another study showed that the activation of Cpx suppresses the synthesis of several bulky, energy-consuming membrane complexes, to decrease the envelope burden under stressful conditions (17). However, our understanding of the molecular mechanism regulating this process is incomplete.

In this study, transcriptome analyses of the A. pleuropneumoniae CpxA/CpxR TCS provided evidence that CpxA/CpxR regulates multiple genes under heat stress, including pilus genes and chaperones. All of the chaperone genes, including *dnaK*, *htpG*, *groEL*, *clpB*, *dnaJ*, and *groES*, were previously shown to be upregulated in the Δ*cpxAR* mutant compared with the WT strain. However, this did not explain the mechanism of CpxA/CpxR resistance to heat stress in A. pleuropneumoniae. Here, we showed that CpxA/CpxR suppresses the expression of multiple pilus genes, including several type IV pilus genes, such as *apfA*, *apfB*, *apfC*, *apfD*, *pilM*, *pilN*, *pilO*, and *pilP*. The repression of nonessential protein complexes may relieve the burden on the envelope protein folding machinery, thereby reducing the production of misfolded proteins and helping to conserve finite cellular resources during times of stress (18). Among these pilus genes, we found that only type IV pilus gene *apfA* was crucial for CpxA/CpxR-mediated heat stress in A. pleuropneumoniae. Together, these observations may suggest that the function of the Cpx response is tightly linked to the ApfA protein content.

**FIG 5:**
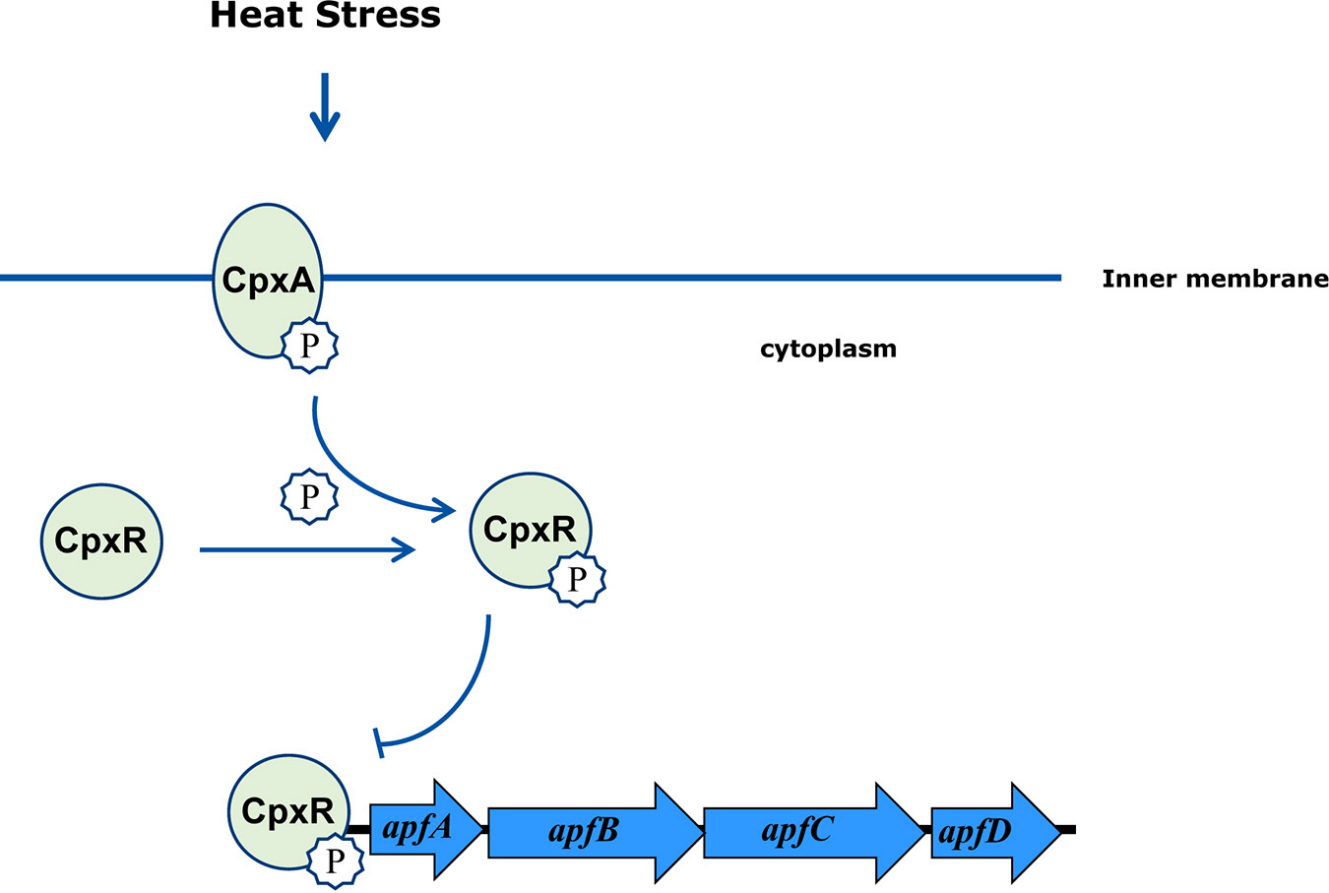
Model illustrating that CpxA/CpxR mediates heat resistance in A. pleuropneumoniae. Under heat stress, CpxA/CpxR responds by undergoing autophosphorylation and directly repressing the transcription of the *apf* operon.

Type IV pili, including type IVa and IVb pili, are dynamic surface appendages present in many Gram-negative and Gram-positive bacteria (19). A previous study showed that Cpx pathway activation tends to inhibit the synthesis of the bundle-forming pili (BFP), the type IVb pili (20). However, whether the type IVa pili are regulated by the Cpx response remains unknown. We showed that phosphorylated CpxR binds directly to the promoter region of the *apfABCD* operon, encoding the pilin subunit ApfA and repressing its transcription. These results revealed the synergistic mechanism between CpxA/CpxR and ApfA under heat stress, whereby CpxR directly suppresses the expression of ApfA (Fig. 5). The stoichiometry of the CpxR-*apfA* complex seems to change at a higher CpxR concentration, possibly due to dimerization of the protein. A. pleuropneumoniae infection in swine can cause a febrile systemic disease involving a body temperature increase of up to 42°C. Further studies will focus on the role of the CpxA/CpxR-ApfA pathway in pathogenesis *in vivo*.

## MATERIALS AND METHODS

### Strains, media, and plasmids.

The strains, plasmids, and primers used in this study are detailed in Tables S1 and S2. A. pleuropneumoniae strains were grown in Trypticase soy broth (TSB) medium (Difco Laboratories, USA) supplemented with 10 μg/mL NAD (BioFroxx, China) and 10% (vol/vol) newborn calf serum (Every Green, China). Escherichia coli strains were cultured in LB medium at 37°C. E. coli β2155, used for transconjugation, was grown in LB supplemented with 50 μg/mL diaminopimelic acid (DAP; Sigma-Aldrich, USA).

### Construction of mutant and complementary strains.

The *apf* and *pil* gene deletion mutants were constructed based on the *ΔcpxAR* mutant by the method of homologous recombination using the suicide vector pEMOC2 as described previously (12). The complementation strain *ΔcpxAR*-CΔ*apfA* was constructed using the E. coli-A. pleuropneumoniae shuttle vector pJFF224-XN as described previously (9). Each correct mutant and complementary strain was verified by PCR amplification (Fig. S1), reverse transcription-PCR (RT-PCR), amplification, and DNA sequencing (data not shown).

### RNA extraction and RNA-seq methods.

The WT (S4074) and *ΔcpxAR* mutant strains were grown in TSB overnight and then transferred (at a 1:1,000 dilution) into fresh medium and grown to an optical density at 600 nm (OD_600_) of 0.6 at 37°C or 42°C. Total RNA was extracted from the bacterial solution using a Bacteria Total RNA isolation kit (Sangon Biotech, China) following the manufacturer’s protocol. The quality of each RNA sample was examined using an Bioanalyzer 2100 (Agilent Technologies, USA). The qualified samples were sent to Novogene Bioinformatics Technology Co., Ltd., (Beijing, China), for RNA sequencing on an Illumina HiSeq 2000 platform. Analysis of differential expression genes was performed using DESeq R (version 1.18.0). Genes with a log_2_ fold change of >1.0 or a log_2_ fold change of <–1.0 and *P* values of <0.05 found by DESeq were assigned as differentially expressed. Each sample was analyzed using three independent biological replicates.

### Real-time PCR (RT-qPCR).

Total RNA was reverse-transcribed to cDNA using the PrimeScript RT reagent kit with gDNA Eraser (Vazyme, China) according to the manufacturer’s instructions. The mRNA level of the target gene was quantified by RT-qPCR using ChamQ SYBR qPCR master mix (Vazyme). The reaction was performed using the ViiA-7 real-time PCR system (Applied Biosystems, USA) under the following conditions: 5 min at 50°C and then 5 min at 95°C, followed by 40 cycles of 10 s at 95°C and 35 s at 60°C. The results were normalized to A. pleuropneumoniae 16S rRNA and analyzed via the 2^−ΔΔ^*^CT^* method (21). Reverse transcription-PCR (RT-PCR) was carried out as described previously (22). The PCR products were electrophoresed and photographed using an ImageQuant LAS 4000 system (GE Healthcare, New Jersey, USA).

### Electrophoretic mobility shift assay (EMSA).

The expression vector pET-28a-cpxR was constructed, and the recombinant protein CpxR was obtained and phosphorylated according to a previously reported procedure (12). The promoter regions of the *apfA and pilM* genes were amplified by PCR and labeled by terminal deoxynucleotidyl transferase (TdT) using an EMSA probe biotin labeling kit (Beyotime, China), generating biotin-labeled probes. The binding of His-CpxR to the biotin-labeled probes was performed using a chemiluminescent EMSA kit (Beyotime) according to the procedure recommended by the manufacturer. Samples were loaded onto a 4% nondenaturing polyacrylamide gel buffered with 0.5× Tris-borate-EDTA (TBE) and blotted onto nylon membrane (Beyotime). Visualization was performed using the chemiluminescent EMSA kit (Beyotime).

### DNase I footprinting assay.

DNase I footprinting experiments were performed using a 6-carboxyfluorescein (FAM)-labeled primer according to a previously described method (23). Briefly, the DNA probe containing the *apfA* promoter region was amplified using the primer pair M13R-48 (FAM)/M13F-47 from the carrier PUC-19 and subsequently purified using the Wizard SV gel and PCR clean-up system (Promega, USA). For each assay, approximately 400 ng probe was incubated with or without 10 μg CpxR protein in a total volume of 40 μL in EMSA buffer at 25°C for 30 min; 10 μL solution containing about 0.015 unit DNase I (Promega) and 100 nmol freshly prepared CaCl_2_ was added, and further incubation was performed at 37°C for 1 min. Following termination by DNase I stop solution, samples were extracted with phenol-chloroform and dissolved in 30 μL of double-distilled water (ddH_2_O). The results were analyzed using the GeneScan-LIZ600 size standard (Applied Biosystems).

### Spotting assays.

Spotting experiments were carried out as described previously (24). A. pleuropneumoniae S4074 and its mutant derivatives were grown to the exponential phase (0.6 at OD_600_) at 37°C. Then, 5 μL of 10-fold serial dilutions were spotted onto TSB plates and incubated at 37°C or 42°C for 36 h before growth was assessed.

### Viability measurements.

The viability of WT strain S4074 and its mutant derivatives after treatment at different temperatures was assessed using the LIVE/DEAD BacLight bacterial viability kit (Invitrogen) according to the manufacturer’s instructions. The propidium iodide (PI) red fluorescent stain labeled the dead cell population of A. pleuropneumoniae, and the SYTO 9 green fluorescent stain labeled the viable cells. A confocal laser scanning microscope (FV1000; Olympus) was used to visualize changes in the viability of A. pleuropneumoniae, and the live and dead microorganism populations of A. pleuropneumoniae were analyzed using the BD FACSCanto II flow cytometer (Becton, Dickinson) and BD FACSDiva 7.0 software.

### Statistical analysis.

Prism software (version 6.0; GraphPad, La Jolla, CA) was used for all statistical analyses. All data are presented as the mean ± standard deviation. Results were evaluated by analysis of variance (ANOVA). *P* values of <0.05 were considered significant (*).
