# Supplementary material for: CpxAR of Actinobacillus pleuropneumoniae Contributes to Heat Stress Response by Repressing Expression of Type IV Pilus Gene apfA
Source: Microbiol Spectr. 2022 Oct 19;10(6):e02523-22. doi: 10.1128/spectrum.02523-22 (PMC9769684; doi:10.1128/spectrum.02523-22)
Supplement: Supplemental file 1 — Fig. S1; Tables S1 and S2. Download spectrum.02523-22-s0001.pdf, PDF file, 0.4 MB [file spectrum.02523-22-s0001.pdf]

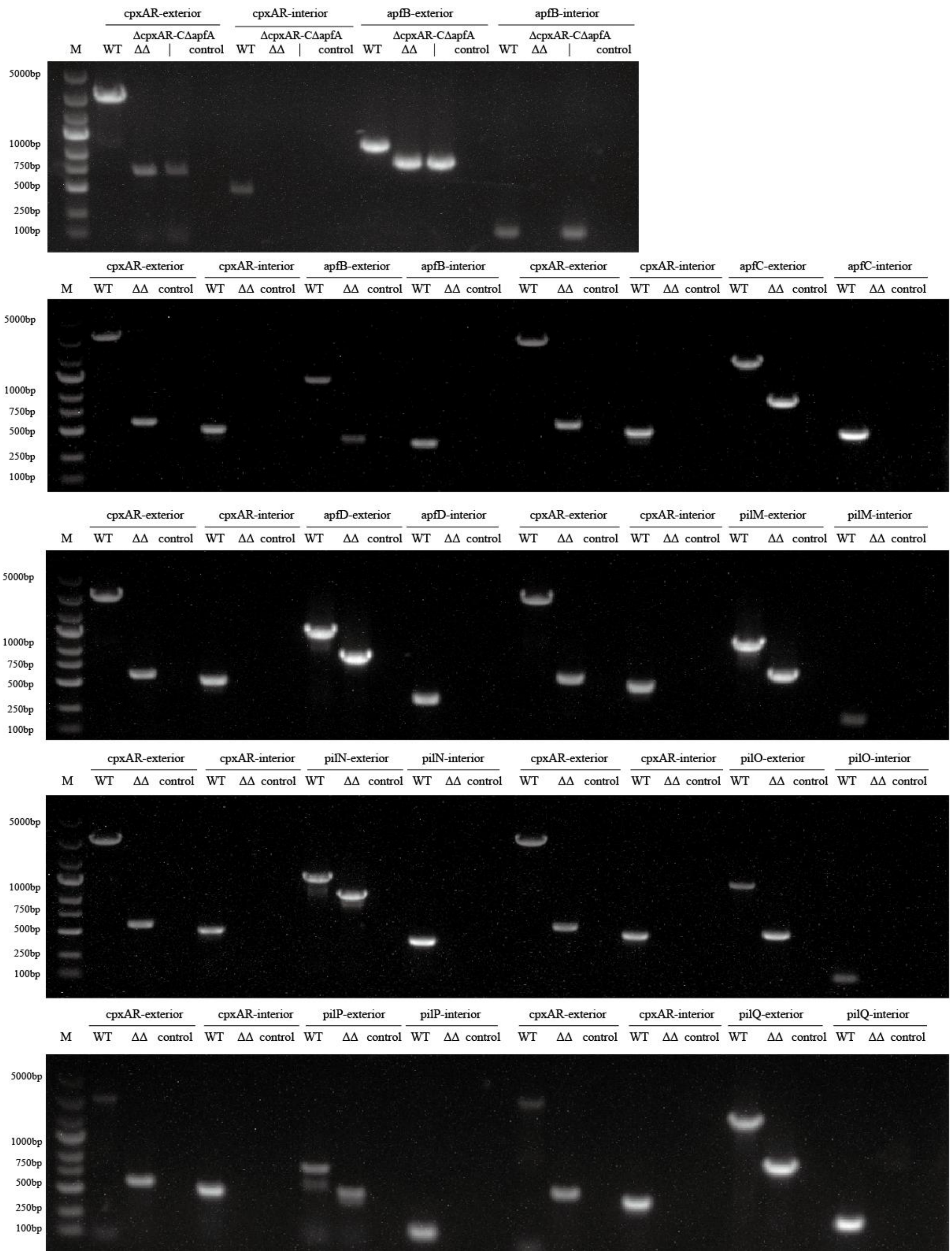

Supplementary Fig. 1 The *A. pleuropneumoniae* mutant and complemented strain verification. Identification of Δ*cpxAR*Δ*apfA*, Δ*cpxAR*Δ*apfB*, Δ*cpxAR*Δ*apfC*, Δ*cpxAR*Δ*apfD*, Δ*cpxAR*Δ*pilM*, Δ*cpxAR*Δ*pilN*, Δ*cpxAR*Δ*pilO*, Δ*cpxAR*Δ*pilP*, Δ*cpxAR*Δ*pilQ* mutant strains and the Δ*cpxAR*-CΔ*apfA* complemented strain by PCR, using PCR primer pairs exterior F/R and interior F/R.

Table S1 Bacterial strains and plasmids used in this study

Strains/plasmids	Characteristics	Source/reference
	A. pleuropneumoniae	
S4074	*A. pleuropneumoniae* reference strain of serovar 1; WT strain	Dr P. Blackall
ΔcpxAR	A. pleuropneumoniae 4074 *cpxAR*-deletion mutant	Laboratory
ΔcpxARΔapfA	A. pleuropneumoniae 4074 *cpxAR/apfA*-deletion mutant	This study
ΔcpxARΔapfB	A. pleuropneumoniae 4074 *cpxAR/apfB*-deletion mutant	This study
ΔcpxARΔapfC	A. pleuropneumoniae 4074 *cpxAR/apfC*-deletion mutant	This study
ΔcpxARΔapfD	A. pleuropneumoniae 4074 *cpxAR/apfD*-deletion mutant	This study
ΔcpxARΔpilM	A. pleuropneumoniae 4074 *cpxAR/pilM*-deletion mutant	Laboratory
ΔcpxARΔpilN	A. pleuropneumoniae 4074 *cpxAR/pilN*-deletion mutant	This study
ΔcpxARΔpilO	A. pleuropneumoniae 4074 *cpxAR/pilO*-deletion mutant	This study
ΔcpxARΔpilP	A. pleuropneumoniae 4074 *cpxAR/pilP*-deletion mutant	This study
ΔcpxARΔpilQ	A. pleuropneumoniae 4074 *cpxAR/pilQ*-deletion mutant	This study
CΔcpxAR	Complemented strain of *ΔcpxAR*; Cm^r	Laboratory
E. coli		
DH5a	Cloning host for recombinant vector	Takara
β2155	Transconjugation donor for constructing mutant strain	From Prof. Gerald-F. Gerlach
	Plasmid	
pEMOC2	Transconjugation vector: ColE1 ori mob RP4 sacB, Amp^rCm^r	From Prof. Gerald-F.

		Gerlach
pEΔ*apfA*	Up- and down-stream arms of *apfA* were ligated sequentially into pEMOC2, and used as the transconjugation vector for *apfA* gene deletion	Our Laboratory
pEΔ*apfB*	Up- and down-stream arms of *apfB* were ligated sequentially into pEMOC2, and used as the transconjugation vector for *apfB* gene deletion	Our Laboratory
pEΔ*apfC*	Up- and down-stream arms of *apfC* were ligated sequentially into pEMOC2, and used as the transconjugation vector for *apfC* gene deletion	Our Laboratory
pEΔ*apfD*	Up- and down-stream arms of *apfD* were ligated sequentially into pEMOC2, and used as the transconjugation vector for *apfD* gene deletion	Our Laboratory
pEΔ*pilN*	Up- and down-stream arms of *pilN* were ligated sequentially into pEMOC2, and used as the transconjugation vector for *pilN* gene deletion	Our Laboratory
pEΔ*pilO*	Up- and down-stream arms of *pilO* were ligated sequentially into pEMOC2, and used as the transconjugation vector for *pilO* gene deletion	Our Laboratory
pEΔ*pilP*	Up- and down-stream arms of *pilP* were ligated sequentially into pEMOC2, and used as the transconjugation vector for *pilP* gene deletion	Our Laboratory
pEΔ*pilQ*	Up- and down-stream arms of *pilQ* were ligated sequentially into pEMOC2, and used as the transconjugation vector for *pilQ* gene deletion	Our Laboratory
pEΔ*pilQ*	Up- and down-stream arms of *pilQ* were ligated sequentially into pEMOC2, and used as the transconjugation vector for *pilQ* gene deletion	This study

Table S2 Primers used in this study

Primer	Sequence (5′–3′) a	Use
cpxAR-W-F	CGAACTTACGCTGACG	detection exterior of *cpxAR* mutants
cpxAR-W-R	ATGGCGCAATACCCT	detection exterior of *cpxAR* mutants
cpxAR-N-F	CAGTGTAATAGCAAGTAAGATAGCG	detection exterior of *cpxAR* mutants
cpxAR-N-R	GTCTCCGGAAGAAAATAGCAA	detection exterior of *cpxAR* mutants
apfA-W-F	AAAGCAAAAATCTCGCAAGCACTGA	detection exterior of *apfA* mutants
apfA-W-R	CGCCACTTGTTGTAGGGATTCTTCA	detection exterior of *apfA* mutants
apfA-N-F	TTTGACCGCCGCTACAGTTTTT	detection interior of *apfA* mutants
apfA-N-R	GTGATTGCGATTATTGCCATTT	detection interior of *apfA* mutants
apfB-W-F	GATAAACGCCGATTCTGCCTCTGT	detection exterior of *apfB* mutants
apfB-W-R	TCATAACGGTAGCACTATTTCTTGG	detection exterior of *apfB* mutants
apfB-N-F	CCGAAGGCGCATCATTGGTATG	detection interior of *apfB* mutants
apfB-N-R	TCTTGGCGGAGCAGAAGGAAAA	detection interior of *apfB* mutants
apfC-W-F	CAAAACAAAGACCGCAGCAACATAG	detection exterior of *apfC* mutants
apfC-W-R	CATAATCCAAACGCAAATAAACCCG	detection exterior of *apfC* mutants
apfC-N-F	ATCTGCGCTACATTCCCACTCTGTT	detection interior of *apfC* mutants
apfC-N-R	GCTTTCTCTCGCCATGCTCCTTTTT	detection interior of *apfC* mutants
apfD-W-F	CGACTGTCGTTTTGCCACTTCC	detection exterior of *apfD* mutants
apfD-W-R	GCTTTCTCTCGCCATGCTCCTT	detection exterior of *apfD* mutants

apfD-N-F	GAGAATAAGGGCGAGAGAGCGA	detection interior of *apfD* mutants
apfD-N-R	AGCCGAAATATAAATGGTGGGG	detection interior of *apfD* mutants
pilM-W-F	ACCTTGGGTATCGCCTTGACTGGA	detection exterior of *pilM* mutants
pilM-W-R	GGCAAGACCTATCGCAACGCTCAC	detection exterior of *pilM* mutants
pilM-N-F	AGGGAAACTTATTGGCAACTCA	detection interior of *pilM* mutants
pilM-N-R	GGAAAGCATTGGTAATCGAAAT	detection interior of *pilM* mutants
pilN-W-F	GGCTTATCGATGCTTTTCCTTT	detection exterior of *pilN* mutants
pilN-W-R	ATTTTTCCTGTTCCAGTTCCCA	detection exterior of *pilN* mutants
pilN-N-F	GAGCGTTGCGATAGGTCTTGCC	detection interior of *pilN* mutants
pilN-N-R	CGATTTCTTGTTGTTCATTCAG	detection interior of *pilN* mutants
pilO-W-F	CCGTTCAAACCTTTACTAAGAGCCA	detection exterior of *pilO* mutants
pilO-W-R	TTCATCTGCCAGCCAATCATTCACT	detection exterior of *pilO* mutants
pilO-N-F	CGAGTCTTATTCTCACCAAGAGTTA	detection interior of *pilO* mutants
pilO-N-R	TTTTTCCTGTTCCAGTTCCCATTGC	detection interior of *pilO* mutants
pilP-W-F	TTTTCAGTCACTCATCAAGTGTCGA	detection exterior of *pilP* mutants
pilP-W-R	TATTGCCGTAGCCGCTTTCTCATCT	detection exterior of *pilP* mutants
pilP-N-F	ATCTCATTACCACAAATTTCCCGTT	detection interior of *pilP* mutants
pilP-N-R	TTCATCTGCCAGCCAATCATTCACT	detection interior of *pilP* mutants
pilQ-W-F	TTTATCGGCGTGTTGAAAAAAGAGG	detection exterior of *pilQ* mutants
pilQ-W-R	CGGAAATAGACGGAAGAAAATGGGT	detection exterior of *pilQ* mutants

pilQ-N-F	GCCAGCCCTCGTTTATTAACCACCA	detection interior of *pilQ* mutants
pilQ-N-R	ATCGGCACTCTGTCCTCACCTTTTG	detection interior of *pilQ* mutants
ApfA-HF-F	GGCTCGAGATGCAAAAACTAAGTCTTATTCGAC	amplification of apfA
ApfA-HF-R	TTGCGGCCGCTTAATTTGATGCGCAGAAATTTGCC	amplification of apfA
apfA-F	GACCGCCGCTACAGTTTTTA	detection the transcription of *apfA*
apfA-R	TTGCCATTTTAGCTACGGTTG	detection the transcription of *apfA*
apfB-F	GATCTTCGGCGGTCAAAATA	detection the transcription of *apfB*
apfB-R	CGGAGCAGAAGGAAAAGTTG	detection the transcription of *apfB*
apfC-F	TCCGCCTAAATCAAAAATCG	detection the transcription of *apfC*
apfC-R	TGCGAGTGTGTTCGGTACTC	detection the transcription of *apfC*
apfD-F	TAAGGGCGAGAGAGCGATAA	detection the transcription of *apfD*
apfD-R	GTTGCTGCGGTCTTTGTTTT	detection the transcription of *apfD*
pilM-F	CGCCCATATGGTGAAAAAATTCGCCGAATCGTTCA	detection the transcription of *pilM*
pilM-R	CGCCCTCGAGTTATTCCTCGCCATTCCATAAACTC	detection the transcription of *pilM*
pilN-F	ATGGAATGGCGAGGAATAAA	detection the transcription of *pilN*
pilN-R	CAAGCGGTCTGTTTTGTTGA	detection the transcription of *pilN*
pilO-F	CCGACAACGATTAACGGAAG	detection the transcription of *pilO*
pilO-R	AGTTCCCATTGCAGCTGTTC	detection the transcription of *pilO*
pilP-F	AAACCCAACGTTTTCAGCAG	detection the transcription of *pilP*
pilP-R	CAGTTGCTTTTGAGCATCCA	detection the transcription of *pilP*

pilQ-F	CGATTGTGCTAGGCGGTATT	detection the transcription of *pilQ*
pilQ-R	GTGAGGAAATTTGCGGTTGT	detection the transcription of *pilQ*
apfA-EMSA-F	GTTTTCGCTAACCAGTAAGATGA	amplification of *apfA* promoter region for EMSA
apfA-EMSA-F	ACGGTATTCCAAGACTTACATTC	amplification of *apfA* promoter region for EMSA
pilM-EMSA-F	CGGCGGCGATGCCACCTA	amplification of *pilM* promoter region for EMSA
pilM-EMSA-F	AAGCGGTTTTTTTAACATTTTTGGC	amplification of *pilM* promoter region for EMSA
rpoE-EMSA-F	TAAAAAGATAAGATAAGCGGTC	amplification of *rpoE* promoter region for EMSA
rpoE-EMSA-R	AGTGTGTAACAAAAATGAAAAGT	amplification of *rpoE* promoter region for EMSA
rpoD-EMSA-F	GCGGAAGAAAAGCAAGAGTTGGTCA	amplification of *rpoD* promoter region for EMSA
rpoD-EMSA-R	TCCATAATTGTATCCGTTTTGTGTG	amplification of *rpoD* promoter region for EMSA
